# Issues with Modeling a Tunnel Communication Channel through a Plasma Sheath

**DOI:** 10.3390/s22010398

**Published:** 2022-01-05

**Authors:** Anna V. Bogatskaya, Andrey E. Schegolev, Nikolay V. Klenov, Evgeniy M. Lobov, Maxim V. Tereshonok, Alexander M. Popov

**Affiliations:** 1 Faculty of Physics, Lomonosov Moscow State University, 119991 Moscow, Russia; annabogatskaya@gmail.com (A.V.B.); alexander.m.popov@gmail.com (A.M.P.); 2 P N Lebedev Physical Institute, Russian Academy of Sciences, 119991 Moscow, Russia; 3 Skobeltsyn Institute of Nuclear Physics, Lomonosov Moscow State University, 119991 Moscow, Russia; tanuior@gmail.com; 4 Science and Research Department, Moscow Technical University of Communication and Informatics, 111024 Moscow, Russia; Lobovrts@yandex.ru

**Keywords:** blackout problem, electromagnetic wave tunneling, numerical modeling, waves in plasma, re-entry spacecraft radio line

## Abstract

We consider two of the most relevant problems that arise when modeling the properties of a tunnel radio communication channel through a plasma layer. First, we studied the case of the oblique incidence of electromagnetic waves on a layer of ionized gas for two wave polarizations. The resonator parameters that provide signal reception at a wide solid angle were found. We also took into account the unavoidable presence of a protective layer between the plasma and the resonator, as well as the conducting elements of the antenna system in the dielectric itself. This provides the first complete simulation for a tunnel communication channel. Noise immunity and communication range studies were conducted for a prospective spacecraft radio line.

## 1. Introduction

A communication channel through the plasma sheath can solve the blackout problem for re-entry of spacecrafts [1,2,3,4,5,6,7,8,9,10,11]. The essence of this problem is that a supersonic flying vehicle in the atmosphere is inevitably covered by a weakly conducting ionized gas, which is opaque for electromagnetic waves in the frequency range 100 MHz–10 GHz (the most important range for current telecommunications). The tunnel communication channel method remains one of the most promising solutions [12]. The implementation consists of covering of the antenna surface with a dielectric layer that will act as a resonator (potential well in terms of quantum-optical analogy) and provide effective tunneling of the signal through the plasma for input radiation frequencies coinciding with the eigen resonator frequencies [12,13,14].

But will the proposed concept work in practice? To answer this question, it is necessary to simulate the transmission of telemetry data through the proposed tunnel communication channel. To carry out the simulation under consideration, we have to improve the model of interaction of electromagnetic waves with the plasma sheath in order to analyze the case of an arbitrary polarization and incident angle. We also have to find a way to take into account the presence of an antenna in the dielectric layer of the considered resonator.

As a result, the structure of the article is as follows. In Section 2, we describe a theoretical model for analyzing the incidence of so-called transverse electric (TE) and transverse magnetic (TM) waves on the plasma layer around the re-entry spacecraft. We show how to achieve signal transmission even at large angles of incidence on the plasma sheath. In Section 3, a mathematical model for a resonator with a bolometric antenna covered with an additional protective layer is presented. Here, we determine the amplitude–frequency and phase–frequency responses of the proposed receiving system. In Section 4, we discuss the results of modeling for data transmission through a tunnel communication channel.

## 2. Resonant Tunneling at Oblique Incidence

**Figure 1:**
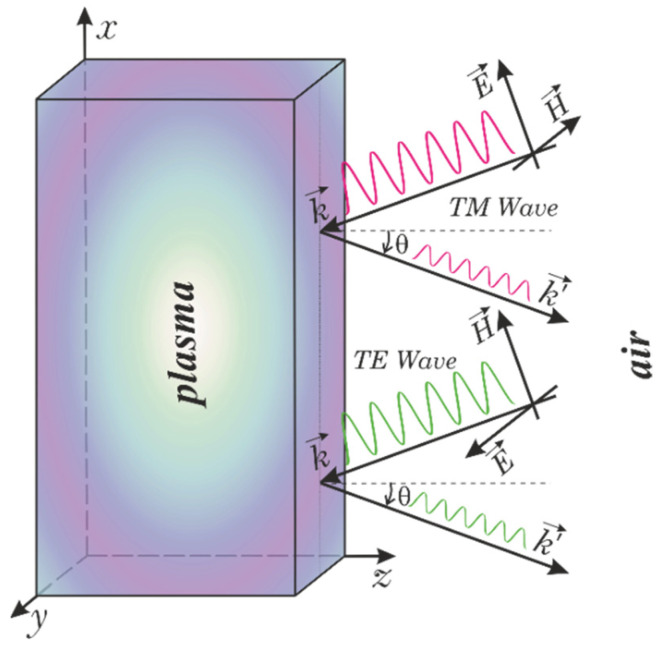
Schematic illustration of the oblique incidence of the electromagnetic wave components (TE and TM waves) on the spacecraft surface covered by a plasma sheath.

Preliminary calculations [12] have shown that by changing the angle of incidence of electromagnetic waves on the plasma surface, one can reduce the transmission of radio signals through the tunnel communication channel by almost an order of magnitude. Here, we plan to discuss the possibility to obtain a more stable signal for different angles of incidence. Let us consider the oblique incidence of the electromagnetic wave on the vehicle surface. In this case, one can distinguish two types of electromagnetic waves: transverse electric (TE), when the electric field vector is parallel to the vehicle surface covered by the plasma sheath, and transverse magnetic (TM), when the same is valid for the magnetic field vector (see Figure 1). Definitely, for the normal wave incidence, the results for these two cases coincide with each other.

We start with the case of the TE wave. Here, we assume that all the layers are lying in the *xy*-plane, the wave vector lies in the *xz*-plane, and *θ* is the angle of incidence of the electromagnetic wave measured from the *z*-axis (Figure 1). In this case, the electric field has the only a tangential *y*-component, while for the magnetic field, both *x-* and *z*-components are non-zero. For the electric field component, one should solve the following equation [15]:

(1) $$\frac{\partial^{2}E_{\omega }}{\partial x^{2}}+\frac{\partial^{2}E_{\omega }}{\partial z^{2}}+\varepsilon \left(z\right)\frac{\omega^{2}}{c^{2}}E_{\omega }=0$$

where ε(z) represents the permittivity profile of considered structure:

(2) $$\varepsilon \left(z\right)=\{\begin{array}{c} \varepsilon_{d},\;0\leq z\leq a\\ \varepsilon_{p},\;a<z\leq a+d\\ \varepsilon_{air},\;z>a+d \end{array}$$

Here, *a* is the thickness of the dielectric layer (acting as a resonator) with permittivity $\varepsilon_{d}>>1$, *d* and *ε_d_* are the same parameters for the plasma sheath (typically *d* = 5 – 10 cm, plasma density $n_{e}=10^{10}÷10^{11}$ cm^−3^) and $\varepsilon_{air}=1$ is the permittivity of the atmospheric air.

As concerns the TM polarization of waves, it turns out to be more convenient to consider the Helmholtz equation for the magnetic field component:

(3) $$\frac{\partial^{2}H_{\omega }}{\partial x^{2}}+\frac{\partial^{2}H_{\omega }}{\partial z^{2}}+\varepsilon \left(z\right)\frac{\omega^{2}}{c^{2}}H_{\omega }=0$$

Obviously, (3) provides the solutions for the *H*-field component in the TM wave similar to those for *E*-field component in the TE wave. Then, the structure of TM wave electric field can be found from Maxwell’s equations:

(4) $${\vec{E}}_{\omega }=\frac{ic}{\omega \varepsilon \left(z\right)}rot\;{\vec{H}}_{\omega }=\frac{ic}{\omega \varepsilon \left(z\right)}\left[{\vec{e}}_{x}\left(-\frac{\partial H_{\omega }}{\partial z}\right)+{\vec{e}}_{z}\left(\frac{\partial H_{\omega }}{\partial x}\right)\right]$$

One should notice the specifics of the above expression consisting of the presence of *ε(z)* in the denominator, which in the case of the considered dielectric medium, leads to differences in representing the boundary conditions for TM and TE waves. We are going to show (in discussion) that such a feature leads to the difference in TE and TM wave tunneling for both polarizations. This effect is beyond the scope of the optical–mechanical analogy used in [12] to analyze the radio-communication blackout problem. In turn, in the case of a magnetic medium, such specificity will be observed for the TE wave.

For a quantitative description of the electromagnetic signal tunneling through the plasma sheath, the filling factor F(ω) was introduced in [12]:

(5) $$F\left(\omega \right)=max\left\{{\left|E_{\omega }^{\left(d\right)}\right|}^{2}/{\left|E_{\omega }^{\left(0\right)}\right|}^{2}\right\}$$

In (5), $E_{\omega }^{\left(0\right)}$ is the given amplitude of the incident wave field, and $E_{\omega }^{\left(d\right)}$ is the amplitude of the wave field stored in the dielectric resonator. As mentioned above, this dependence is characterized by the sharp maxima at the resonant frequencies. A similar definition for the filling factor is used for the TE and TM waves.

Generally, for wave incidents at some angle to the vehicle surface, besides the energy flux perpendicular to the surface, there will be a flux propagating alongside it. The latter reduces the effectiveness of the resonant transmission and shifts the resonance position to higher frequencies according to the expression:

(6) $$\omega_{n}\approx \frac{\pi c}{a\sqrt{\varepsilon_{d}}cos\phi }n$$

where *φ* is the angle of the slope of the electromagnetic wave in the dielectric layer: $\sin\varphi =\sin\theta \sqrt{\varepsilon_{air}/\varepsilon_{d}}$. It can be seen that embedded dielectric with a high value of permittivity provides resonant frequency stabilization: the greater the ratio $\varepsilon_{d}/\varepsilon_{air}$, the closer the angle *φ* to the zero value.

The wave Equations (1) and (3) were solved numerically as described in [12]. The plasma permittivity was chosen in the form

(7) $$\varepsilon_{p}=1-\frac{\omega_{p}^{2}}{\omega^{2}+\nu_{tr}^{2}}+i\frac{\omega_{p}^{2}\nu_{tr}}{\left(\omega^{2}+\nu_{tr}^{2}\right)\omega }$$

where $\omega_{p}^{2}=4\pi e^{2}n_{e}/m$ is the plasma frequency squared and $\nu_{tr}$ is the transport collisional frequency.

Below, we provide the results of the calculations of the filling factor for the cases of TE, TM, and arbitrary (half the sum of TE and TM) wave polarizations. To demonstrate the specific features of TE and TM wave tunneling, we assume that the thickness *d* of a plasma layer is 10 cm with electron density *n_e_* = 10^11^ cm^−3^ (the plasma frequency is $\omega_{p}^{}\approx 1.78\times 10^{10}$ s^−1^) and *ν _tr_* = 10^8^ s^−1^. The surface of the re-entry spacecraft is covered by the dielectric layer of thickness *a* = 0.50 cm and permittivity *ε_d_* = 700, which correspond to novel materials, in particular to barium titanate [16,17]. For tunnel radio communication channel, the telemetric signal frequency should coincide with one of the eigen-frequencies of the resonator (dielectric layer). In nonresonant cases, the wave field is reflected from the plasma layer and filling is negligible. We have already shown [12] that for the analysis of really important channel features we can consider that the plasma layer is characterized by a rectangular profile of the electron density and the spacecraft surface is ideally conductive.

**Figure 2:**
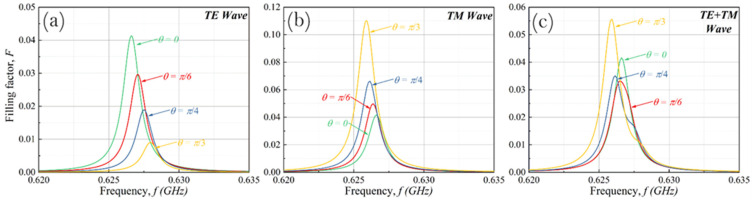
Filling factor for the first resonance at normal and angular incidence for TE (**a**), TM (**b**), and mixed wave polarization (**c**). Dielectric layer parameters: *ε_d_* = 700, *a* = 0.5 cm; plasma sheath parameters: *n_e_* = 10^11^ cm^–3^, *ν_tr_* = 10^8^ s^–1^, *d* = 10 cm.

The obtained data for the filling factor in dependence on f=ω/2π near one of the resonances are presented in Figure 2. First, it can be seen that for considered dielectric parameters, the resonance shifting is rather small (within the resonance bandwidth). Further, the decreasing of its maximum value is observed. The last circumstance appears due to the decrease of the normal component of the incoming electromagnetic wave flux. As a result, the greater the angle of incidence, the greater the resonance shifting (see Equation (6)). Nevertheless, the dielectric layer with a high value of permittivity plays the role of a «stabilizer», preventing significant displacement of resonant peaks. For *ε_d_* = 700, which is within the range of particular interest for the problem under discussion, the resonance shifting is negligible. For almost any angle of incidence, the wave in the resonator propagates nearly normally to the vehicle surface. Comparing data in Figure 2a,b one can notice that, unlike the TE wave, the filling factor for the TM wave grows slightly with an angle increase, and the resonant position shifts slightly to the left. In particular, this effect is associated with the direction of the electric field strength vector. Due to the presence of the *E*-field component normal to the surface, the TM wave interacts more efficiently with the plasma layer. As a result, better signal tunneling is observed near the resonance frequency region. From a practical point of view, both the angle of incidence and the contribution of the TE and TM components can be varied during the signal transferring to the vehicle. This means that the filling factor should be calculated for mixed polarization. It is important to notice that for the case of mixed polarization, the differences between the TE and TM cases partially compensate each other: for the case of the equal contribution of both polarizations (such a case corresponds to the unpolarized GHz pulse), the data presented in Figure 2c demonstrate a very weak filling factor dependence on the angle of wave incidence.

## 3. Accounting for a Protective Layer and Antenna Elements

**Figure 3:**
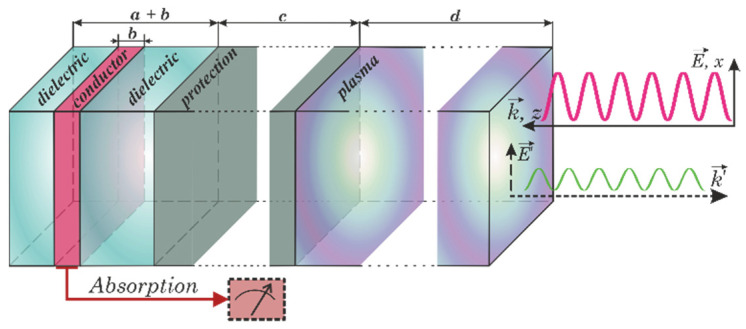
Sketch of the dielectric resonator with protection and additional metal layer inside playing the role of the receiving antenna element.

In this chapter, we will make our study more specific. First, as the dielectric resonator is of a rather small size, we will study the bolometric signal detection. In this case, the thin conducting layer inside the resonator serves as a part of the receiving antenna. Second, we will take into account the additional heat-resistant dielectric layer that protects the resonator from plasma heating. Both additional layers can contribute to the eigen-mode frequencies and field distribution in the structure. The characteristics of the resonator considered above give rise to the possibility to study the case of the normal incidence of the electromagnetic wave only. Our structure under investigation is shown in Figure 3.

The following parameters were introduced in the model: *a* and *ε_d_* are the total thickness and permittivity of the dielectric layer, *b* and *z_res_* are the thickness and position of the conducting layer, $N_{e}$ and *ν* are the electronic concentration and transport frequency in the conducting layer, and *c* and *ε_c_* are the thickness and permittivity of the protecting layer.

Further, we will neglect the possible electromagnetic loss in all the dielectric layers. For considered permittivity and the desired frequency range, one can obtain the possible range of the dielectric layer thickness. The thickness of the protective layer was chosen for reasons of compactness and efficiency, and the materials were selected from among those already in operation for these purposes.

As the first step, we investigated field modes inside the dielectric layer without a metal layer. For the given parameters of the structure, there were three eigen-frequencies in the resonator below the plasma frequency. It can be seen that due to a wide protecting layer, the distribution of the field in the modes behaves as if in a shallow well of width *a*. Further, we mainly analyze the tunneling channel at the frequency of the third mode of the resonator (*f* = 2.47 GHz). In this case, the electromagnetic field packet passing through the plasma undergoes the fastest decay [14].

**Figure 4:**
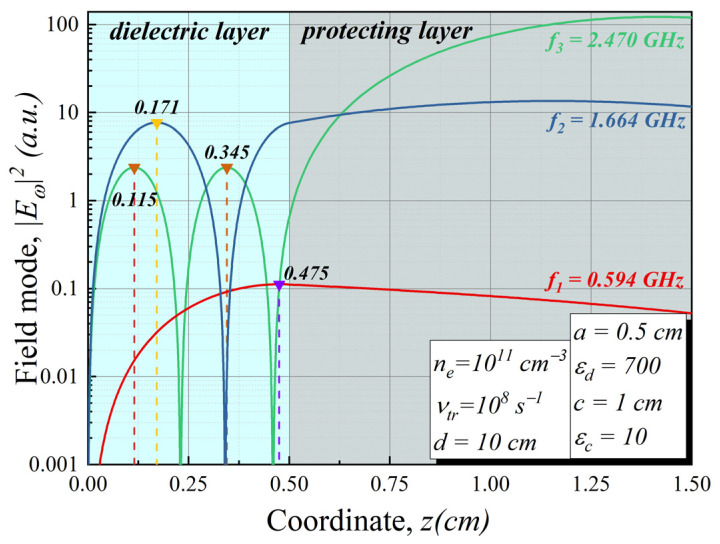
Mode distributions inside the structure (no metal layer). Parameters used: *a* = 0.5 cm, *ε_d_* = 700, *c* = 1 cm, *ε_c_* = 10, *d* = 10 cm.

**Figure 5:**
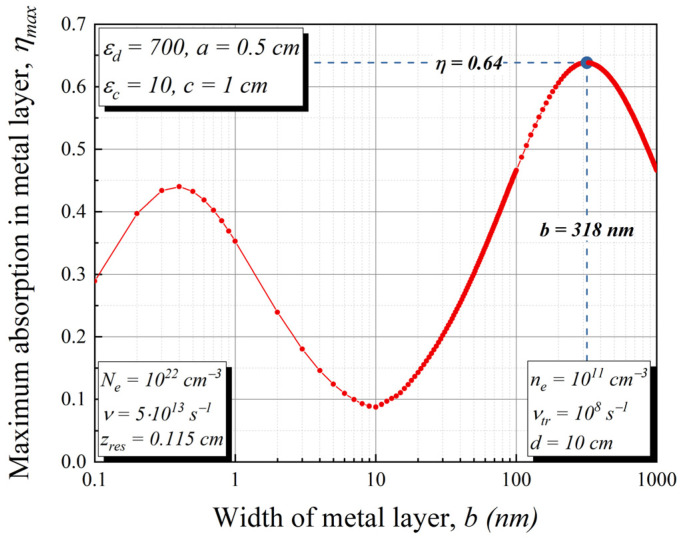
Dependence of maximum absorption *η* in the metal layer on the width of this metal layer *b.* The parameters are *a* = 0.5 cm, *ε_d_* = 700, $z_{res}=0.115$ cm, *c* = 1 cm, *ε_c_* = 10, *d* = 10 cm.

**Figure 6:**
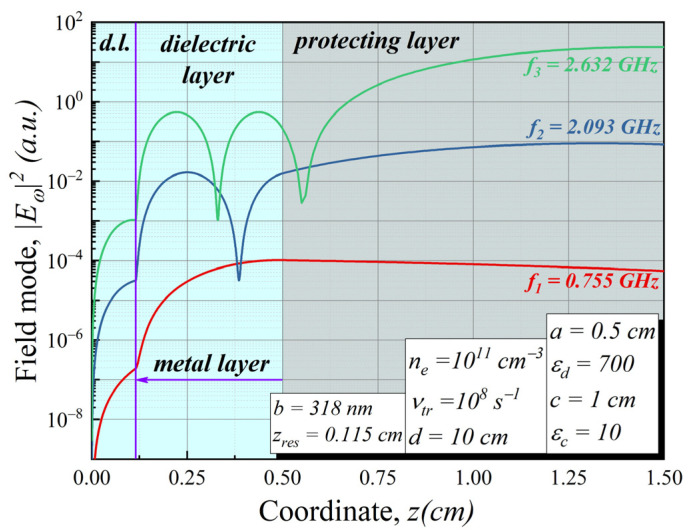
Modes distribution inside the considered Figure 3 structure. Different colors schematically illustrate the structure’s layers. Parameters used: *a* = 0.5 cm, *ε_d_* = 700, *b* = 318 nm, *z_res_* = 0.115 cm, *c* = 1 cm, *ε_c_* = 10, *d* = 10 cm.

The fraction of the flux absorbed by the thin conductive layer can be calculated as follows [18]:

(8) $$\eta_{\omega }=\frac{1}{cI_{\omega }^{2}}\frac{\omega_{p}^{2}\nu }{\omega^{2}+v^{2}}\int E_{\omega }^{2}\left(z\right)dz$$

where $I_{\omega }$ is the intensity of the incident wave of frequency ω, $E_{\omega }$ is the spatial field distribution, and the integral is taken over the area inside the conducting layer, $z\in \left(z_{res},b+z_{res}\right)$. Numerical calculations have shown that the metal layer of the parametric antenna in a relatively thin dielectric resonator changes the structure of the field, and for the optimal absorption, we chose *z_res_* = 0.115. The final absorption weakly depends on the value of $z_{res}$. It is much more interesting to consider the dependence of the absorption on the width of the metal layer, *b*. An example for *z_res_* = 0.115 cm is shown in Figure 4. There are two local maxima (see Figure 5): the first one corresponds to the value *b* = 0.4 nm with $\eta_{\omega }\approx 0.44$ and the second to the value *b* = 318 nm with $\eta_{\omega }\approx 0.64$. The reason for this non-monotonic dependence is that the metal layer divides the resonator into two parts with its own standing waves. By gradually changing the value of *b*, we change the boundary conditions for each of the modes, obtaining the maximum absorption at the “new” resonance. For further consideration, we will select the last set of parameters (*z_res_* = 0.115 cm; *b* = 318 nm) for the metal layer used as part of the bolometric detector with sufficiently strong signal absorption, see Figure 6 for example.

**Figure 7:**
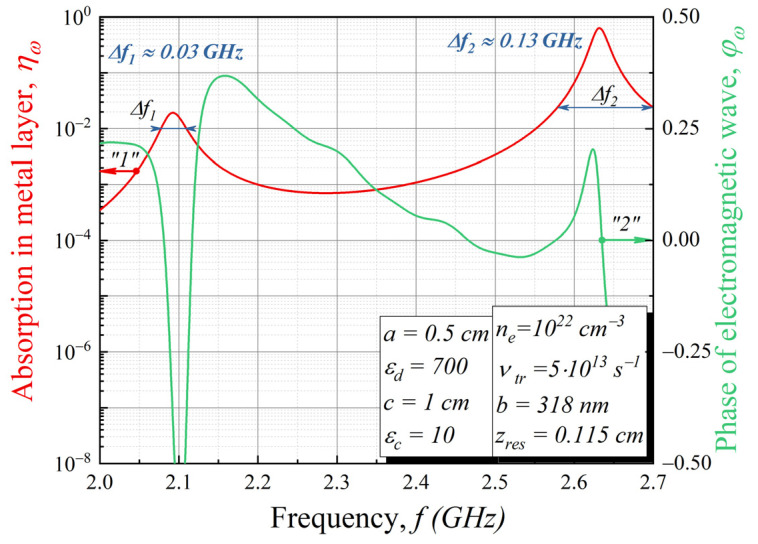
Amplitude–frequency and Phase–frequency responses of the structure shown in Figure 3 for the following parameters: *a* = 0.5 cm, *ε_d_* = 700, *b* = 318 nm, *z_res_* = 0.115 cm, *c* = 1 cm, *ε_c_* = 10, *d* = 10 cm.

The main characteristics of the considered radio technical device are the amplitude–frequency response (AFR) and phase–frequency response (PFR). In our case, the frequency dependence of absorbed energy in the metal layer plays the role of the AFR (“1” line in Figure 7), while the frequency dependence of phase shift of incident and reflected waves represents the PFR (“2” line in Figure 7). To obtain the PFR response, we provide a reminder that the reflection from the structure field is given by the expression

$$E_{\omega }\left(z\right)=E_{0}\;exp(-ikz)+B_{\omega }\;exp(ikz)$$

where the complex amplitude of the reflected wave is $B_{\omega }=B_{0}\exp(i\varphi_{\omega })$ and k=ω/c. Taking into account that φω=atan(Im(Bω)/Re(Bω)), we have:

(9) $$\varphi_{\omega }\left(z\right)=\mathrm{atan}\left\{\frac{Im\left[E_{\omega }\left(z\right)\;exp(-ikz)/E_{\omega }^{\left(0\right)}-\;exp(-2ikz)\right]}{Re\left[E_{\omega }\left(z\right)exp(-ikz)/E_{\omega }^{\left(0\right)}-\;exp(-2ikz)\right]}\right\}$$

There are two peaks of absorption in the frequency range 2.0 GHz–2.7 GHz (2.7 GHz ≈ (2*π*)^−1^*ω_p_*). The first peak is rather small (*η_ω_* ≈ 0.02) with central frequency 2.093 GHz. The second peak has *η_ω_* ≈ 0.64 with a central frequency 2.632 GHz and Δ*f*_2_ = 130 MHz on the level of absorption of 0.025. The absorption scale was graduated from $10^{-8}$ to 1, where the ground level corresponds to the case when there is almost no absorption of the external electric field in the metal layer, and “level 1” corresponds to the full absorption of the external electric field. Therefore, up to 64% of an external wave field can be absorbed at the carrier frequency ~2.6 GHz.

## 4. Simulations of the Tunnel Radio Communication Channel

A numerical simulation of a prospective telemetric and control radio line for a spacecraft was performed to analyze the efficiency of overcoming blackout. Optimal reception of radio signals is usually performed using matched filtering [19]. The frequency response of a matched filter directly depends on the spectral density of the signal and noise.

**Figure 8:**
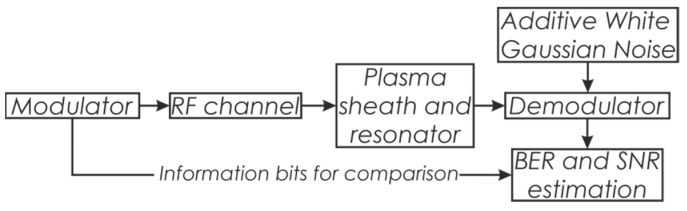
Simulation scheme for the tunnel communication channel.

The plasma sheath and resonator distort the radio signal due to the absorption of signal energy in certain frequency ranges. The amplitude–frequency and phase–frequency response curves of the “plasma + resonator” system are depicted in Figure 7. It is seen that the system’s frequency response is quite uneven. The matched filter was designed as a finite impulse response (FIR) filter with tap coefficients representing a complex-conjugate impulse response of the “plasma + resonator” system. The numerical simulation of the radio signal reception via the proposed resonator was conducted using the scheme depicted in Figure 8. An RF channel was implemented as signal attenuation due to energy loss in the atmosphere. Signal energy loss was calculated using the ITU-R P.618-13 recommendation taking into account the loss caused by signal propagation in the atmosphere, the influence of clouds, and precipitation for the 2.632 GHz operating frequency. The considered system, consisting of the plasma sheath and resonator, was implemented as the FIR filter with tap coefficients representing the impulse response of the “plasma + resonator” system. The modulator was designed to form a binary shift-keying (BPSK) signal with a direct sequence spread spectrum (DSSS).

The spreading sequence is a maximum-length sequence (MLS). The demodulator was designed using a coherent scheme. The model has been used to conduct a noise immunity investigation for signal-code constructions that meet telemetric and control radio line requirements. For this purpose, estimates for the bit error rate (BER) and signal-to-noise ratio (SNR) were included in the model (that is very common for a variety of tasks) [20,21,22,23,24,25]. SNR was estimated as the ratio of the spectral power density in the band of the signal to the spectral power density outside this band. BER was estimated as a ratio of the number of incorrectly decoded bits to the total number of transmitted bits. The signal properties are presented below in Table 1.

**Figure 9:**
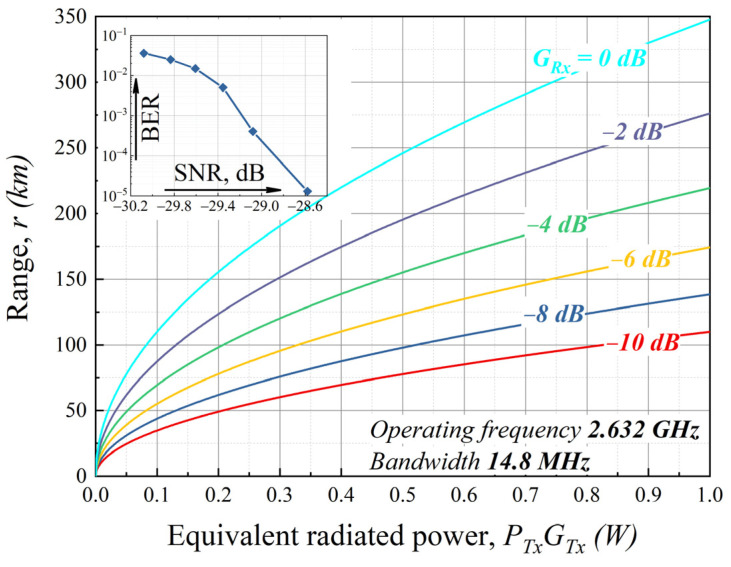
Communication range through the tunnel communication channel at operating frequency 2.632 GHz and bandwidth 14.8 MHz.

According to ITU-R P.618-13, the average atmospheric loss for the 2.632 GHz operating frequency is about 3 dB. However, more than 3 dB will be lost due to the imperfect synchronization scheme operation and Doppler effect compensation according to the navigation system data. The calculations lead us to the curves of the maximum communication range depending on the equivalent radiated power *P_Tx_ G_Tx_*, where *G_Tx_* is gain of the transmitting antenna and *P_Tx_* is radiated power. These curves are depicted in Figure 9. Each curve represents a given receiving antenna gain *G_Rx_*.

Figure 9 shows that the communication range can reach 350 km using a 1 Watt transmitter and usual compact antennas, which is more than enough for communication with the space vehicle [26,27,28].

## 5. Conclusions

In conclusion, we would like to note that our research shows the efficiency of the proposed resonator. The efficiency varies across the required bandwidth (the loss due to resonator imperfection is 0.7 dB in the 14.8 MHz band and 0.44 dB in the 7.4 MHz band). We have achieved this due to solutions of two key issues of a real tunnel communication channel through a plasma sheath. (1) We have analyzed the incidence of electromagnetic waves at an arbitrary angle. (2) We have taken into account the presence of an antenna in a dielectric resonator. Thus, the proposed resonator provides an extensive communication range, making telemetry and control transmissions possible during the entire flight of spacecrafts. We have prepared a plan for a set of laboratory experiments to validate the proposed method.

## Figures and Tables

**Table 1:** Signal Properties and Channel Parameters in Simulations.

Property/Parameter	Value
Signal type	BPSK with DSSS
Pseudo-random sequence length	1024
Error-correcting code	convolutional turbo code, r = 1/3
Bandwidth, MHz	14.8
Chip rate, MBd	7.4
Net data rate, kbit/s	2.2
Operating frequency, GHz	2.632
Receiver antenna gain, dB	0 to −10
Receiver noise factor, dB	2
Maximum receiver temperature, °C	100
Receiver bandwidth, MHz	14.8
Required SNR in the receiving band	−28.5 (BER = 10^−4^)

## Data Availability

Not applicable.
